# Association Between Juvenile Psychotic Experiences and Problematic Gaming

**DOI:** 10.1093/schizbullopen/sgae021

**Published:** 2024-09-02

**Authors:** André Fernandes, Renan M Biokino, Andrew C C Miguel, Viviane Machado, Gabriela Koga, Laís Fonseca, Pedro M Pan, Thiago Henrique Roza, Giovanni Salum, Ives Cavalcante Passos, Luis Augusto Rohde, Euripedes Constantino Miguel, Carolina Ziebold, Ary Gadelha

**Affiliations:** Department of Psychiatry, Federal University of São Paulo (Unifesp), São Paulo, Brazil; Department of Psychiatry, Interdisciplinary Laboratory in Clinical Neuroscience (LiNC), Federal University of São Paulo (Unifesp), São Paulo, Brazil; Department of Psychiatry, Federal University of São Paulo (Unifesp), São Paulo, Brazil; Department of Psychiatry, Interdisciplinary Laboratory in Clinical Neuroscience (LiNC), Federal University of São Paulo (Unifesp), São Paulo, Brazil; Department of Psychiatry, Federal University of São Paulo (Unifesp), São Paulo, Brazil; Department of Psychiatry, Interdisciplinary Laboratory in Clinical Neuroscience (LiNC), Federal University of São Paulo (Unifesp), São Paulo, Brazil; Department of Psychiatry, Federal University of São Paulo (Unifesp), São Paulo, Brazil; Department of Psychiatry, Interdisciplinary Laboratory in Clinical Neuroscience (LiNC), Federal University of São Paulo (Unifesp), São Paulo, Brazil; Department of Psychiatry, Federal University of São Paulo (Unifesp), São Paulo, Brazil; Department of Psychiatry, Interdisciplinary Laboratory in Clinical Neuroscience (LiNC), Federal University of São Paulo (Unifesp), São Paulo, Brazil; Department of Psychiatry, Federal University of São Paulo (Unifesp), São Paulo, Brazil; Department of Psychiatry, Interdisciplinary Laboratory in Clinical Neuroscience (LiNC), Federal University of São Paulo (Unifesp), São Paulo, Brazil; Schizophrenia Program (PROESQ), Federal University of São Paulo (Unifesp), São Paulo, Brazil; Department of Psychiatry, Federal University of São Paulo (Unifesp), São Paulo, Brazil; Department of Psychiatry, Interdisciplinary Laboratory in Clinical Neuroscience (LiNC), Federal University of São Paulo (Unifesp), São Paulo, Brazil; National Institute of Developmental Psychiatry (INPD), São Paulo, Brazil; Department of Psychiatry, Federal University of Paraná (UFPR), Curitiba, Brazil; National Institute of Developmental Psychiatry (INPD), São Paulo, Brazil; Department of Psychiatry, Graduate Program in Psychiatry and Behavioral Sciences, Federal University of Rio Grande do Sul (UFRGS), Porto Alegre, Brazil; National Institute of Developmental Psychiatry (INPD), São Paulo, Brazil; Department of Psychiatry, Graduate Program in Psychiatry and Behavioral Sciences, Federal University of Rio Grande do Sul (UFRGS), Porto Alegre, Brazil; Laboratory of Molecular Psychiatry, Experimental Research Center (CPE) and Clinical Research Center (CPC), Hospital de Clínicas de Porto Alegre (HCPA), Porto Alegre, RS, Brazil; National Institute of Developmental Psychiatry (INPD), São Paulo, Brazil; Department of Psychiatry, Graduate Program in Psychiatry and Behavioral Sciences, Federal University of Rio Grande do Sul (UFRGS), Porto Alegre, Brazil; ADHD Outpatient Program and Developmental Psychiatry Program, Hospital de Clínicas de Porto Alegre, Federal University of Rio Grande do Sul (UFRGS), Porto Alegre, Brazil; Medical Council UNIFAJ & UNIMAX, Indaiatuba, Brazil; National Institute of Developmental Psychiatry (INPD), São Paulo, Brazil; Department of Psychiatry, Institute of Psychiatry, University of São Paulo (USP), São Paulo, Brazil; Department of Psychiatry, Federal University of São Paulo (Unifesp), São Paulo, Brazil; Department of Psychiatry, Interdisciplinary Laboratory in Clinical Neuroscience (LiNC), Federal University of São Paulo (Unifesp), São Paulo, Brazil; Department of Psychiatry, Federal University of São Paulo (Unifesp), São Paulo, Brazil; Department of Psychiatry, Interdisciplinary Laboratory in Clinical Neuroscience (LiNC), Federal University of São Paulo (Unifesp), São Paulo, Brazil; Schizophrenia Program (PROESQ), Federal University of São Paulo (Unifesp), São Paulo, Brazil; National Institute of Developmental Psychiatry (INPD), São Paulo, Brazil

**Keywords:** gaming disorder, gaming addiction, addictive behaviors, psychotic experiences, Brazil

## Abstract

**Background and Hypothesis:**

Problematic gaming (PG) is an emerging mental health condition associated with significant adverse outcomes. Even though PG has been linked to other psychiatric disorders, its association with psychotic experiences (PEs) remains poorly explored to date. The aim of our study was to examine the association between both conditions in a large Brazilian community sample. We hypothesized that adolescents with PG were more likely to report PE compared with those without the disorder.

**Study Design:**

Our investigation was based on a cross-sectional subsample of a large Brazilian cohort (*n* = 1616; 13- to 21-year age range). Using the 7-item version of the Game Addiction Scale, participants were classified according to their gaming status: no PG, PG, or gaming addiction (GA). The association between PG, GA, and PE was assessed through linear regression analyses, which were adjusted for the presence of significant covariates, including other psychiatric conditions.

**Study Results:**

9.5% (*n* = 154) presented PG and 2.7% (*n* = 43) had GA. 28.0% received any *Diagnostic and Statistical Manual of Mental Disorders*, Fourth Edition (DSM-IV) diagnosis and the mean PE score was 9.39 (*SD* = 4.35). Participants presenting PG had greater levels of PE, compared with participants with no PG, even controlled by sociodemographic variables and the presence of any DSM-IV diagnosis (*b* = 0.96, 95% CI = 0.17–1.75, *P* = .017).

**Conclusions:**

According to our results, PG was significantly associated with PE, even in the presence of other covariates. Although preliminary, these results suggest that PG and PE may have shared neurobiological and/or behavioral pathways.

## Introduction

Psychotic experiences (PEs) refer to subclinical hallucinations and delusions that are common in the general population, with an estimated prevalence of 17% among children aged 9–12 years and 7.5% in adolescents aged 13–18 years.^1^ Although mostly transitory and benign,^2^ the report of PEs is associated with impairments in social achievement^3^ and occupational functioning,^4^ as well as increased later risk of psychosis and suicidality.^5^

PE has already been associated with neurobiological findings connected to schizophrenia, more specifically, dopamine imbalance and changes to brain salience network connectivity.^6^ This may explain the propensity to develop psychotic disorders as well as addictive behaviors related to reward circuits, such as problematic gaming (PG).^7^ In addition, subjects with psychotic symptoms may be more prone to social isolation, which ultimately may also be associated with solitary leisure activities such as gaming.^8^

Gaming disorder (GD) is an emerging mental health issue, which was introduced in the DSM-5 in 2013^9^ as a condition for further studies and recognized as an official diagnostic category by the World Health Organization (WHO) in the International Classification of Diseases 11th Revision (ICD-11) in 2019.^10^ PG and GD have been associated with several negative outcomes, including distress and impairment in several life contexts, sedentary lifestyle, problems in hygiene and self-care, among others.^11,12^ Furthermore, psychiatric disorders have also been linked to GD, with some presenting significant comorbidity with this addictive behavior.^12^ For instance, social anxiety, depression, and attention-deficit/hyperactivity disorder (ADHD) are some of the conditions reported in the literature to be associated with GD, although the causality direction is uncertain.^13^

However, to date, the relationship between GD and PE has been poorly explored in literature. A recent scoping review highlighted a significant gap in this topic: only 6 case reports and 2 cross-sectional studies were found.^14^ The first cross-sectional study investigated the potential beneficial roles of internet gaming, among 104 patients with schizophrenia, as a coping strategy for self-stigma and psychological distress.^15^ The second study, with parental reporting of GD, found this comorbidity in one of the 11 inpatient psychotic adolescents; no further analysis was conducted.^16^ Another cross-sectional, non–peer-reviewed study found that adolescents and young adults who experienced more PE had a higher probability of reaching the cutoff for GD.^17^

Thus, 2 main pathways were suggested to explain this association. Firstly, both GD and PE can share common pathophysiological mechanisms, as both disorders may involve changes in the salience network and dopamine neurotransmission.^6,7,18^ Secondly, subjects experiencing psychotic or even prodromal symptoms are more likely to be socially withdrawn, spending more time on the internet, social media, or gaming.^8^

Hence, this study aimed to investigate the association between PG and PE in a large Brazilian sample of adolescents and young adults. To the best of our knowledge, this association has not been assessed in a peer-reviewed investigation. Considering the possibility that PG and PE share similar dopaminergic mechanisms, we hypothesized that participants with PG would present higher levels of PE. Furthermore, we explored whether this association remains significant when adjusting for the presence of comorbid psychiatric disorders.

## Methods

### Subjects

We analyzed data from a subsample of the Brazilian High-Risk Cohort Study (BHRCS), a prospective longitudinal cohort of children and adolescents recruited between 2009 and 2010 at public schools from São Paulo and Porto Alegre, both large Brazilian urban centers. Further details on sampling procedures are described elsewhere.^19^ Briefly, 12 500 parents of children aged 6–14, attending 57 schools (22 in Porto Alegre and 35 in São Paulo), were invited to a screening interview using the Family History Screen (FHS),^20^ which assesses all family members for *Diagnostic and Statistical Manual of Mental Disorders*, Fourth Edition (DSM-IV) mental health disorders. Biological parents—with the biological mother being the main informant in 87% of the cases—answered the FHSing for 8012 families, representing 9937 children. An index of the family load was computed for each of the potentially eligible children based on the percentage of members in the family that screened positively for each of the disorders assessed and adjusted for relatedness. High-risk selection targeted those screening positively for any of the 5 main psychiatric diagnoses of interest (ADHD, anxiety disorders, OCD, PEs, and learning disorders). Among these, children with a higher number of family members (biological mother, biological father, biological sibling, or half-sibling) affected were prioritized. The final cohort was comprised of 2511 children (baseline), 957 of which were randomly selected, and 1554 were a subsample of children at increased risk of mental disorders based on the FHS. A total of 2010 school students participated in the first follow-up (9–17 years) and 1905 in the second follow-up (13–21 years). These study data were collected during the psychological interview at the second follow-up (*n* = 1616, years 2018–2019) when an assessment of PG was included in the study protocol.

The whole research was performed in compliance with the Declaration of Helsinki. The Ethics Committees of the Federal University of São Paulo, University of São Paulo, and Hospital de Clínicas de Porto Alegre approved all study-related procedures. Children’s assent and informed consent of the parents or participants aged above 18 years were obtained.

### Materials

#### Assessment of PE (Outcome).

 PEs were evaluated by the Community Assessment of Psychic Experiences—Positive Dimension (CAPE-Pos). CAPE-Pos is a 20-item self-report questionnaire, intended to measure PE, that is stable, reliable, and valid for self-reported psychotic-like experiences in the general population.^21^ Such questionnaire measures psychotic-like symptoms using a 4-point Likert scale to indicate the frequency (0 = never, 1 = rarely, 2 = often, 3 = almost always). The score could then range from 0 to 60. CAPE-Pos can also be divided into the following 3 dimensions: Perceptual Abnormalities dimension, Persecutory Ideation dimension, and Bizarre Experiences dimension.^22^

#### Assessment of Gaming Status (Exposure).

 We measured gaming status with the 7-item version of the 21-item Game Addiction Scale (GAS), developed by Lemmens et al.^23^ The Portuguese version^24^ shows good intrinsic validity (Cronbach’s alpha [α] = .94), a correlation between items, and test–retest validity (α = .92). GAS is a screening tool for addictive game use in the last 6 months (eg, investigating salience—*how often the participants think about playing all day long*; tolerance—*increasing amounts of time on gaming*; mood modification—*play to release stress*; relapse—*failure to reduce game time*; withdrawal—f*eeling bad when unable to play*; conflict—*have fights with others over the time spent*; problems—*neglect other important activities as school*). The 7 items are measured on a 5-point continuum scale: 1 (never), 2 (rarely), 3 (sometimes), 4 (often), and 5 (very often). Total score ranges between 7 and 35, with higher scores indicating a higher level of gaming problems. Following criteria used in previous investigations,^24^ we classified participants according to their gaming status as presenting: (a) A pattern of problematic gaming (PG) when they answered “sometimes” to at least 4 of the 7-item GAS; (b) Gaming addiction (GA) when individuals answered “often” or “very often” to at least 4 items; or (c) No PG.

#### Assessment of Covariates.

 Age (years at assessment), gender (male or female), self-reported skin color (White or Non-White, including Mixed-race, Black, Indigenous, and Asian), State (Rio Grande do Sul or São Paulo), and Family Risk of Psychiatric Disorders (high-risk or not, according to the criteria explained in the sampling procedures) were included as covariates. We also included any psychiatric diagnosis, according to the Brazilian-Portuguese version of the Development and Well-being Assessment (DAWBA), a validated and widely used structured interview applied by lay interviewers in various studies. All questions from DAWBA are closely related to DSM-IV diagnostic criteria and focus on current problems causing significant distress or social impairment.^25,26^ Verbatim responses as well as structured answers are then carefully evaluated by psychiatrists that confirm, refute, or change the initial computerized diagnosis. A total of 9 certified child psychiatrists performed the rating procedures. All of them were trained and supervised closely by a senior child psychiatrist with extensive experience in rating the DAWBA. In addition, all cases in which raters had doubts about any specific diagnosis were scaled up and discussed between 2 child psychiatrists until consensus about the diagnosis was achieved.

### Data Analysis

#### Descriptive Statistics.

 We first explored the distribution of all study variables. Quantile–quantile plots were used to check the normal distribution of continuous variables. Categorical variables are reported by the number of observations and percentages, and continuous variables through means and standard deviations.

#### Missing Data.

 Owing to a programming error on the data collection platform, responses to the last 4 items of the CAPE (corresponding to the Perceptual Abnormalities dimension) were only provided by participants who reported a positive response to the item *“Do you believe in the power of witchcraft, voodoo, or the occult?.”* To correct this issue, we employed multiple imputations to handle the missing data. Specifically, we generated 5 imputed data sets using a logistic regression model that included all available information from the other CAPE items as predictors. After a comprehensive scan of the data, the fully conditional specification technique was determined to be the most appropriate method. We assessed the accuracy of the imputed values using descriptive statistics, histograms, and boxplots. The final analysis reports the combined outcomes from these 5 imputed data sets. We also present a complete case analysis as a sensitivity analysis, including available items from the Persecutory Ideation dimension (9 items) and the Bizarre Experiences dimension (6 items).

#### The Association Between Gaming Status and PE.

 We first conducted bivariate linear regression models where the dependent variable was PE scores, and the predictor was gaming status (no PG, PG, or GA). We then tested a multivariate linear regression model including gaming status as a predictor adjusted for all study covariates, according to the enter method where all variables were forced to be included in the model. Finally, interaction analyses were conducted to evaluate the moderation effect of gaming status on the association between any psychiatric disorder and PE. Also, sensitivity analyses were performed using GAS scores as a continuous variable.

The normal distribution of errors and collinearity was evaluated for the multivariate model. We assumed a significance level of 5% (2-tailed). All analyses were performed using the Statistical Package for the Social Sciences, SPSS, version 24.

## Results

Sociodemographic and clinical characteristics of the sample, overall and by gaming problem status, are shown in table 1. Most participants, 53.5% (*n* = 865), were male, with a mean age of 18.37 years (*SD* = 1.99), 60.9% (*n* = 981) were White, and 51.2% (*n* = 827) were from São Paulo state. Of note, 60.9% (*n* = 981) had a familial risk of psychiatric disorder at recruitment. The average GAS score was 3.51 (*SD* = 4.92), ranging from 0 to 28. According to clinical characteristics, 9.5% (*n* = 154) presented PG and 2.7% (*n* = 43) showed GA. A total of 453 participants (28.0%) received any DSM-IV diagnosis and the mean PE according to CAPE was 9.39 (*SD* = 4.26). Supplementary table S1 shows that the distribution of sociodemographic and clinical baseline characteristics was similar between subjects who were assessed at follow-up and those who were not.

**Table 1.** Sociodemographic and Clinical Characteristics of the Sample, Overall and by Gaming Problem Status (BHRC, *n* = 1616)

Characteristics	No Problematic Gaming		Problematic Gaming		Gaming Addiction		Total	
	*n*	%	*n*	%	*n*	%	*n*	%
Overall	1419	87.8	154	9.5	43	2.7	1616	100
Gender								
Male	697	49.1	133	86.4	35	81.4	865	53.5
Female	722	50.9	21	13.6	8	18.6	751	46.5
State								
Rio Grande do Sul	704	49.6	67	43.5	18	41.9	789	48.8
São Paulo	715	50.4	87	56.5	25	58.1	827	51.2
Skin color								
White	861	60.8	95	61.7	25	58.1	981	60.9
Non-white	554	39.2	59	38.3	18	41.9	631	39.1
Family risk of psychiatric disorder (yes)	861	60.7	106	68.8	25	58.1	992	61.4
Any psychiatric disorder (yes)	381	26.8	53	34.4	19	44.2	453	28.0
	Mean	*SD*	Mean	SD	Mean	SD	Mean	SD
Age	18.4	2.0	18.1	2.0	17.4	2.1	18.4	2.0
Psychotic experiences score	9.2	4.2	10.5	4.6	11.2	4.8	9.4	4.3

**Fig. 1.**
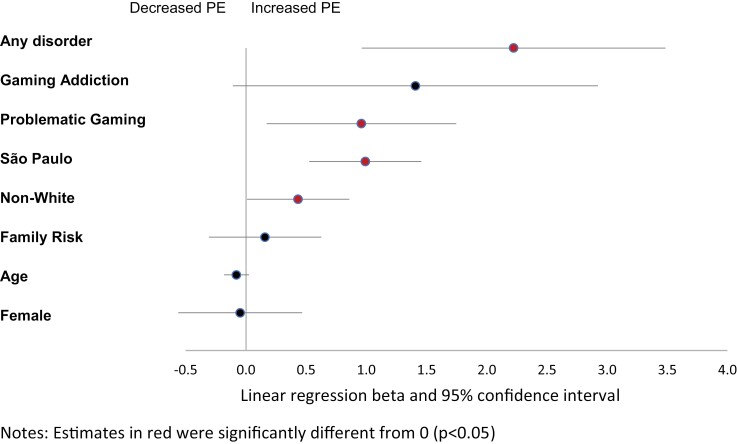
Factors associated with psychotic experiences (PE). Estimates in red were significantly different from 0 (*P* < .05) (color figure online).

Table 2 shows the results of bivariate and multivariate analyses of the association between gaming status (no PG, PG, and GA), covariates, and PE. Bivariate analyses showed that participants presenting gaming problems and GA had greater levels of PE compared with participants without gaming problems. PE levels were also higher among participants presenting any psychiatric disorder and living in São Paulo State. PG remained significantly associated with PE in multivariate analyses (*b* = 0.96, 95% CI = 0.17–1.75, *P* = .017, table 2 and figure 1). None of the covariates had a variance inflation factor greater than 1.10, meaning that multicollinearity was low. PG and PE remained significantly associated in sensitivity analyses using the original (not imputed) data set (*b* = 4.98, 95% CI = 0.64–9.32, *P* = .025).

**Table 2.** Linear Regression Models: Association Between Gaming Status and PE

Predictor	Bivariate Analysis			Multivariate Analysis		
	β	95% CI	*P* value	β	95% CI	*P* value
No Problematic Gaming (Ref.)	[Ref]	[Ref]	[Ref]	[Ref]	[Ref]	[Ref]
Problematic Gaming (PG)	**1.24**	**0.48–2.02**	**.002**	**0.96**	**0.17–1.75**	**.017**
Gaming Addiction (GA)	**1.98**	**0.41–3.54**	**.015**	1.41	–0.11 to 2.92	.068
Covariates						
Age	–0.07	–0.17 to 0.03	.183	–0.08	–0.18 to 0.03	.138
Gender (female)	0.01	–0.56 to 0.59	.965	–0.05	–0.56 to 0.47	.854
State (Sao Paulo)	**0.95**	**0.48–1.44**	**<.001**	**0.99**	**0.53–1.46**	**<.001**
Skin color (Non-White)	0.44	–0.002 to 0.88	.051	**0.43**	**0.01–0.86**	**.045**
Family risk of psychiatric disorder (yes)	0.29	–0.19 to 0.78	.234	0.16	–0.31 to 0.63	.501
Any Psychiatric Disorder (yes)	**1.12**	**0.42–1.82**	**.008**	**2.22**	**0.96–3.49**	**.005**

*Note*: Ref = Reference, significant estimates (*P* < .05) are in bold.

**Fig. 2.**
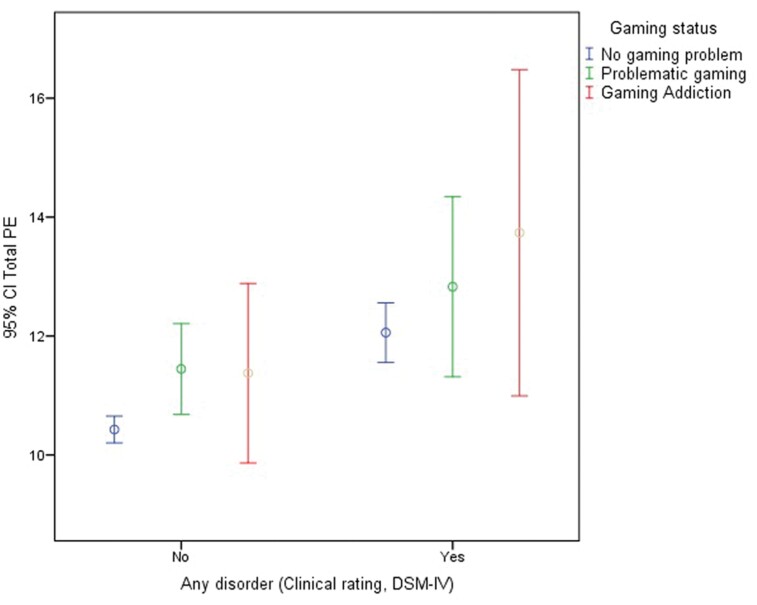
Mean and 95% confidence interval of psychotic experiences (PE) according to comorbidity between any psychiatric disorder and gaming status.

Finally, as table 3 shows, all interactions between PG or GA and any psychiatric disorder significantly predicted higher levels of PE, especially the comorbidity between any psychiatric disorder and GA compared with having only a psychiatric disorder. Figure 2 illustrates the mean differences in PE according to the comorbidity between PG or GA and any other psychiatric disorder.

**Table 3.** Psychotic Experiences Scores Predicted by the Interaction Between Gaming Status and Any Disorder, Adjusted for Covariates

Interaction	β	95% CI	*P* value
No problematic gaming × no disorder (reference)			
No problematic gaming × any disorder	**2.20**	**0.95–3.45**	**.004**
Problematic gaming × no disorder	**1.01**	**0.10–1.92**	**.029**
Problematic gaming × any disorder	**3.07**	**1.06–5.08**	**.006**
Gaming addiction × any disorder	**4.26**	**1.70–6.82**	**.002**

*Note*: Significant interactions (*P* < .05) are in bold.

Supplementary table S2 presents the results of a sensitivity analysis where GAS scores are used as a continuous predictor instead of categorizing gaming status. The analysis indicates that each one-point increase in GAS scores is associated with a 0.07-point increase in PE scores (95% CI = 0.01–0.12, *P* = 0.026), after adjusting for covariates. Complete case analysis using as outcomes the Persecutory Ideation and Bizarre Experience dimension of CAPE-Pos also showed a positive and significant association with GA (Supplementary table S3).

## Discussion

Subjects with PG had a higher score of PE, which remained significant after controlling for potential confounding factors (gender, site, age, and skin color). Also, the presence of a comorbid DSM-IV diagnosis with PG or GA significantly increased the levels of PE. Three main hypotheses could explain this association: (a) as an indirect sign of higher general psychopathology levels; (b) a common neurobiological pathway; or (c) a strategy to deal with the emotional burden linked to PE.

First, in previous studies, PE seems to signal more severe general psychopathology.^4,5^ PG has also been associated with other psychiatric disorders, such as depression, anxiety, and ADHD.^13^ In this perspective, the association between PE and PG could be explained as a sign of more severe psychopathology. To evaluate this possibility, our multivariate model included any psychiatric disorder as a covariate. Our results confirmed the association between PE and general psychopathology, but also underscored that the PG result remained significant despite controlling for such association. Furthermore, we found an interaction between PG and other psychiatric disorders, thus increasing the levels of PE, compared with the presence alone of any—nongaming—disorder.

Second, neuroimaging studies suggest that GD may present similar pathophysiological processes to what is found in other addictive disorders, including a significantly increased salience network connectivity.^7^ Studies indicate that dysregulation of D2 receptors in the orbitofrontal cortex could be associated with the loss of control present in the compulsive behavior of PG, thus explaining similarities with other addictive disorders.^18,27,28^ Under conditions where tonic dopamine activation is increased, salience may also be directed to stimuli that are not objectively important.^29^ The concept of aberrant salience was also introduced to explain hallucinations and delusions, objectively false perceptions, and beliefs in people with schizophrenia.^29^ Moreover, dopaminergic agents such as Levodopa have been linked either to the emergence of psychotic episodes or problematic gambling and other reward-driven behaviors, similar to GD.^30^ A significant co-occurrence of PE and GD may signal dopamine transmission dysregulation between both psychopathological phenotypes.

Third, subjects with PE may experience prodromal symptoms such as social withdrawal, social anxiety, and social anhedonia that may lead these individuals to choose online gaming as a preferred means of social interaction.^17^ Hikikomori, a syndrome characterized by severe social withdrawal, has already been associated with PE^31^ and also with GD.^32^ This piece of evidence suggests that some phenomena could increase both the risk of problematic technology use and PE, such as sensory deprivation, impulsivity, interpersonal deficits, and proneness to magical thinking (causing susceptibility to fantasy games).^31^ Nevertheless, the reasons for playing video games were not investigated in the studies, and asking questions about this sphere in future research can lead us to understand the positive and negative impact of gaming on patient’s life. From a positive perspective, Chang et al^15^ proposed that patients with psychotic disorders could use video games to escape their reality, serving as a coping strategy to decrease depressive and anxiety symptoms, and helping them to connect with others. These authors^15^ speculated that patients could use video games to help them to connect with others, through the perceived anonymity that the internet provides.

In summary, many pathways can explain this association between GD and PE with bidirectional relations. Few previous studies, however, verified this association. In a cross-sectional study, Santesteban-Echarri et al^17^ adapted a compulsive internet use scale to evaluate GD and posted it on online gaming forums. They found that adolescents and young adults who experienced an increased number of PE had a higher probability of reaching the cutoff for a GD. However, these results were from an abstract presented at a congress. Another study, published before the GD concept, found an association between internet addiction and the increase of PE at a 2-month follow-up.^33^

A few limitations must be considered. First, as a cross-sectional study, it is not possible to define the directionality between PG and PE. Second, we had some missing data regarding the PE variable due to a programming error on the data collection platform. Nevertheless, we performed multiple imputations of 5 data sets to handle missing data, and the imputed values were checked. Sensitivity analyses were conducted to evaluate the robustness of our findings across different scenarios, ensuring that the conclusions drawn are as reliable and comprehensive as possible. The results without the imputations were also statistically significant. Third, the outcome of developing psychotic disorders cannot be properly evaluated, since the age of our sample was below the average for the onset of a first psychotic episode.^34,35^ Last, we had a community sample, in which the prevalence of disorders was lower, then our number of participants with PE was small for a few subanalyses.

In conclusion, our study found a statistically significant association between PG and PE in a sample of Brazilian young people. We also found that comorbid GA and any other psychiatric disorders substantially increased the level of PE. Further longitudinal studies are required to better understand the causal relationship between PG and PE. Furthermore, functional imaging studies can help to disentangle common neurobiological and/or behavioral pathways between these 2 phenomena. This could provide a new perspective into vulnerability for disorders such as schizophrenia, during a viable period for early intervention, and even prevention.

## Supplementary Material

Supplementary data are available at *Schizophrenia Bulletin Open* online.

sgae021_suppl_Supplementary_Tables_S1-S3
