# Supplementary material for: Association Between Juvenile Psychotic Experiences and Problematic Gaming
Source: Schizophr Bull Open. 2024 Sep 2;5(1):sgae021. doi: 10.1093/schizbullopen/sgae021 (PMC11408271; doi:10.1093/schizbullopen/sgae021)
Supplement: sgae021_suppl_Supplementary_Tables_S1-S3 [file sgae021_suppl_supplementary_tables_s1-s3.docx]

**SUPPLEMENTARY MATERIAL**

**Table S1. Comparison of sociodemographic and clinical baseline characteristics between subjects who were assessed at follow-up and those who were not (BHRC, n=2511)**

| **Baseline**  **Characteristics** | **Participated at follow-up** | | **Attrition** | | **p value *** |
| --- | --- | --- | --- | --- | --- |
|  | **n** | **%** | **n** | **%** |  |
| **Overall** | 1616 | 64.4 | 895 | 35.6 |  |
| **Male** | 866 | 53.6 | 509 | 56.9 | 0.113 |
| **São Paulo** | 827 | 51.2 | 429 | 47.9 | 0.120 |
| **White** | 981 | 60.9 | 538 | 60.1 | 0.933 |
| **Family risk of psychiatric disorder** | 992 | 61.4 | 561 | 62.7 | 0.522 |
| **Any Psychiatric Disorder** | 430 | 26.6 | 222 | 24.8 | 0.323 |
|  | **Mean** | **SD** | **Mean** | **SD** |  |
| **Age** | 10.18 | 1.89 | 10.23 | 1.92 | 0.443 |

*Chi-square test for categorical variables, T-test for age.

**Table S2. Linear regression models: Association between Gaming Addiction Scale scores and psychotic-like experiences**

| *Predictor* | b | 95% CI | p value |
| --- | --- | --- | --- |
| GAS score | **0.07** | **0.01-0.12** | **0.026** |
| *Covariates* |  |  |  |
| Age | -0.07 | -0.18-0.03 | 0.184 |
| Gender (Female) | 0.02 | -0.55-0.60 | 0.939 |
| State (Sao Paulo) | **0.94** | **0.48-1.40** | **<0.001** |
| Skin Color (Non-White) | **0.44** | **0.01-0.86** | **0.045** |
| Family risk of psychiatric disorder (yes) | 0.18 | -0.29-0.67 | 0.453 |
| Any Psychiatric Disorder (yes) | **2.21** | **0.96-3.46** | **0.005** |

GAS: Gaming Addiction Scale

**Table S3. Linear regression models using complete case analysis: Association between gaming status and Bizarre Experiences and Persecutory Ideation dimension of the Community Assessment of Psychic Experiences - Positive Dimension (CAPE-Pos)**

|  | Persecutory Ideation | | | | Bizarre Experiences | | |
| --- | --- | --- | --- | --- | --- | --- | --- |
| *Predictor* | **b** | | **95% CI** | **p value** | **b** | **95% CI** | **p value** |
| No problematic Gaming | (Reference) (Reference) | | | | | | |
| Problematic Gaming | 0.91 | 1.45-10.77 | | 0.001 | 0.36 | 0.16-1.04 | 0.003 |
| Gaming Addiction | 1.42 | 2.37-8.58 | | 0.003 | 0.66 | 1.07-9.77 | 0.002 |

*Sex, age, state, skin color, family risk of psychiatric disorder and any disorder were included as covariates.
